# Efficacy and Effectiveness of the ProSomnus® [IA] Sleep Device for the Treatment of Obstructive Sleep Apnea: EFFECTS Study

**DOI:** 10.7759/cureus.15391

**Published:** 2021-06-02

**Authors:** Jordan Stern, Kiwon Lee, Dave Kuhns, Jesse F Martinez-Kratz

**Affiliations:** 1 Otolaryngology, Icahn School of Medicine at Mount Sinai, New York, USA; 2 Sleep Medicine, BlueSleep, New York, USA; 3 Dentistry, BlueSleep, New York, USA; 4 Sleep Apnea, ProSomnus Sleep Technologies, Pleasanton, USA; 5 Emergency Medicine, School of Public Health, University of Michigan, Ann Arbor, USA

**Keywords:** sleep apnea, snoring, mandibular advancement devices, oat, cpap, compliance

## Abstract

Objectives

To evaluate the effectiveness of a new mandibular advancement device (MAD) (Prosomnus® [IA] Sleep Device, Prosomnus Sleep Technologies, Pleasanton CA) fitted with a compliance tracker as a first-line treatment in a population of patients with mild to severe obstructive sleep apnea (OSA).

Methods

Treatment effectiveness was measured using pre and post-treatment home sleep testing (HST) and validated sleep and quality of life questionnaires. Mean disease alleviation (MDA) was calculated to compare the treatment effectiveness of MAD to historical continuous positive airway pressure (CPAP) effectiveness data.

Results

MAD was found to be an effective first-line treatment for patients with mild, moderate, and severe sleep apnea with excellent compliance rates, similar to or better than CPAP, and an equal or better MDA of 56.7% compared to literature values of 50% for CPAP.

Conclusions

MAD should be considered an effective first-line treatment for patients with mild and moderate sleep apnea and for severe sleep apnea for patients who prefer, refuse, or are not candidates for CPAP.

## Introduction

Obstructive sleep apnea (OSA) is a common disorder that affects nearly 1 billion people globally [1]. Despite being of epidemic proportions, OSA remains largely undiagnosed with several primary treatment options, including continuous positive air pressure (CPAP), mandibular advancement device (MAD) therapy, surgery, and weight loss [2]. Each treatment modality has variable proven success among patients, with varying levels of disease severity, however, the ratio of the prescribed treatments is nearly 10 to one in favor of CPAP over MAD and other non-CPAP treatments [3-4]. Patients using CPAP often struggle with adherence to the prescribed treatment, with compliance levels after one year ranging from 30%-60% depending on how compliance is defined [5-8]. One definition of compliance with therapy (CPAP) is the use of therapy for at least four hours per night for 70% of nights or approximately five days per week. The question is, whether this is sufficient to reduce the signs and symptoms associated with OSA? The SAVE study (Sleep Apnea cardioVascular Endpoints study) showed no significant reduction in cardiovascular events when patients were treated with CPAP; however, it was observed that the average CPAP use during the study was only 3.3 hrs/night [9]. Weaver demonstrated that the hours of CPAP use are correlated with outcomes [8]; however, Rotenberg concluded that CPAP adherence remains persistently low over 20 years of reported data and thus questions CPAP as the “standard” treatment for obstructive sleep apnea [5].

With the prevalence of OSA increasing year after year, and the advancements in technology for MAD improving the comfort and durability of the devices, there is a need to be able to align the patient preferences with the best and most appropriate therapy. Patients diagnosed with mild, moderate, or severe sleep apnea should be given a choice of therapy that includes MADs. Comparison of the two therapies has been a challenge until recently because there was no method to track compliance with MAD treatment. Recently, sensors embedded in the MAD have enabled the objective measurement of patients’ usage of their device. Mean disease alleviation (MDA), a method for comparing the two therapies, was introduced by Grote and Vanderveken [10-11]. MDA is calculated by taking into consideration both the efficacy of treatment and the actual compliance with treatment. Understanding the mean disease alleviation of a treatment type can help clinicians better evaluate the effectiveness of treatments for OSA while giving patients a choice of therapies rather than limiting it to just one modality - CPAP. This study focuses on the use of MADs as first-line treatment for mild-moderate and severe sleep apnea in a group of patients who have chosen MAD as treatment and refused treatment with CPAP or surgery.

This study was presented as a poster at the annual meeting of the American Academy of Dental Sleep Medicine, Baltimore, Maryland, June 2018.

## Materials and methods

Patients who presented to a specialized sleep apnea diagnostic and treatment center (BlueSleep Center, New York City, NY) [12] and were subsequently diagnosed with obstructive sleep apnea and chose an oral appliance as therapy over CPAP as first-line treatment were offered participation in the trial. Adult subjects were selected from all potential patients when the apnea-hypopnea index (AHI) was equal to or less than 50, with the exclusion of pregnant women (AHI less than 5 is normal, 5-14.9 is mild, 15-29.9 is moderate, and 30 and above is considered severe). Within the study group, patients’ age ranged from 18-75. Initially, 42 subjects consented to participate in the study but only 28 subjects followed through to obtain their MAD and complete post-titration sleep studies. Of the population, 79% were male, with a mean population age of 44.7 years ± 9.8 and a mean body mass index of 28.8 ± 5.3 kg/m^2^. Eleven patients had mild sleep apnea, 11 patients had moderate sleep apnea, and six patients had severe sleep apnea. Population demographics and quality of life (QOL) scores are detailed in Table 1. Institutional review board (IRB) approval was obtained, and informed consent obtained from all participants.

**Table 1:** Patient profiles AHI: Apnea-Hypopnea Index; BMI: Body Mass Index; PSQI: Pittsburgh Sleep Quality Index; Insomnia Severity Index: Insomnia Severity Index; FOSQ: Functional Outcomes of Sleep Questionnaire

Gender	AHI (PRE)	BMI (PRE)	PSQI (PRE)	ISI (PRE)	FOSQ (PRE)
22 Males	21.9 +/- 12.6	27.1 +/- 3.4	6.9+/-4.6	11.7 +/- 6.2	15.3 +/- 3.2
6 Females	21.5 +/- 11.0	34.9 +/- 6.8	6.8 +/- 4.0	13.3 +/- 7.2	14.8 +/- 4.5

**Figure 1:**
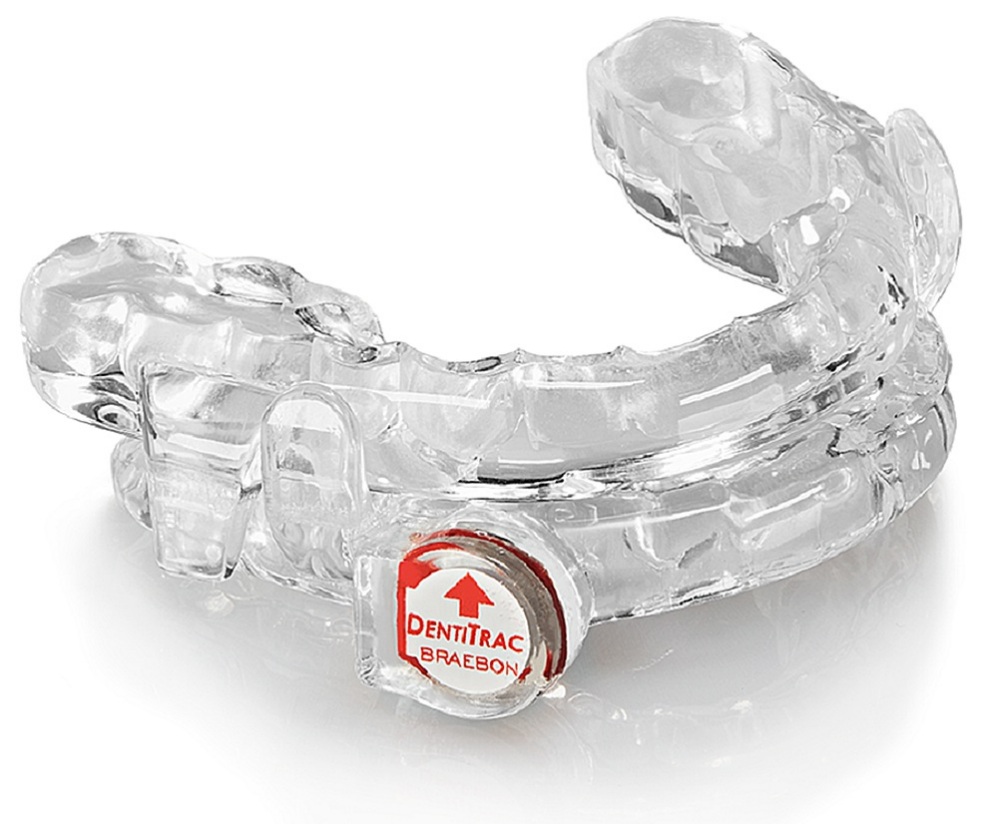
Prosomnus [IA] mandibular advancement device with BRAEBON DentiTrac Prosomnus [IA] mandibular advancement device: Prosomnus Sleep Technologies, Pleasanton, CA BRAEBON DentiTrac: BRAEBON Medical Corporation, Kanata, Ontario, Canada

Participants were treated with the Prosomnus [IA] MAD (Prosomnus Sleep Technologies, Pleasanton CA) with an embedded tracking chip (Dentitrac, BRAEBON Medical Corporation, Ontario, Canada) [13] to track compliance. The MAD consists of a fixed, custom-made lower tray, which has the embedded chip, and three upper trays allowing for 1 mm incremental mandibular protrusion for each tray, or a maximal protrusion of 3 mm total, each tray protruding the mandible 1 mm further than the starting position (Figure 1).

A minimum adherence time of four hours per night and use of at least five out of seven nights a week was considered to be compliant with treatment to align with current Medicare requirements for compliance with CPAP. Efficacy was measured by comparing the average AHI on posttreatment HST to the average pretreatment baseline AHI. Additionally, treatment effectiveness was measured by evaluating pretreatment and posttreatment QOL indices; including the Pittsburgh Sleep Quality Index (PSQI) [14], the Functional Outcomes of Sleep (FOSQ) [15], and the Insomnia Severity Index (ISI). Snoring was evaluated using the Snore Severity Score (SSS) [16]. Actual sleep time was estimated using patient reports. Patients were given home sleep tests (HSTs) using the Alice Night One (Koninklijke Philips N.V., Amsterdam, Netherlands) [17] in replicated two to three nights pretreatment and two to three nights posttreatment after final titration of the MAD. Patients were treated with the ProSomnus [IA] that was fitted with the Dentitrac compliance chip (Figure 1).

MADs are fabricated from dental impressions and the starting protrusion point is based on a comfortable protrusion position for each individual subject. The patients were instructed to change trays every week for three weeks until they reached the prescribed protrusion range. Adherence was calculated on the four hr/night, five out of seven days/week, the standard for CPAP.

## Results

Of the 28 subjects who completed the study, the pretreatment mean AHI was 21.8 ± 12.1 per hour (ranging from 6 to 49) with a posttreatment mean AHI of 8.2 ± 5.9, ranging from 1.2 to 14.5. MDA was calculated as the product of the AHI percent reduction after treatment and the overall compliance rate (as a percentage of oral appliance use on average per night) (Table 2).

**Table 2:** Patient overall outcomes AHI: apnea-hypopnea index; MDA: mean disease alleviation

Population	Initial AHI	POST AHI	Average % Reduction	Overall Compliance	MDA
Total population (100%)	21.8	8.2	54.6%	93.6%	51.1%
% Responders (89.3%)	22.9	7.3	63.9%	93.4%	59.7%
50 % or greater Response (75.9%)	26.9	7.2	72.6%	98.0%	71.1%

Results were also calculated for patients grouped into categories consisting of mild, moderate, and severe sleep apnea. The average reduction in AHI was 74.0% for cases of severe OSA, 72.30% for cases of moderate OSA, and 62.99% for cases of mild OSA (Table 3). The mean reduction in AHI was 13.5 ± 11.7 (Table 4). Patients were considered responders to treatment if their posttreatment AHI decreased compared to pretreatment levels, and this study found that 89.3% of the patients were considered responders to treatment, with an average AHI reduction of 63.9% (Table 2). A 50% or greater reduction in AHI was also calculated and occurred in 75.9% of the study population.

**Table 3:** Response to treatment and mean disease alleviation scores, based on the severity of AHI and response (overall response vs. greater than 50% decrease in AHI) AHI: apnea-hypopnea index; MDA: mean disease alleviation

Severity/Population	Initial AHI average	Post AHI	Average % Reduction	Overall Compliance	MDA
Severe (AHI>= 30), n=6	38.7	9.9	74.0%	100.0%	74.0%
Moderate (AHI >= 15), n=11	22.5	9.9	55.4%	93.8%	52.0%
Mild (AHI < 15), n=11	10.3	5.7	41.4%	87.9%	36.4%
Severe (AHI >= 30) with greater than 50% reduction in AHI	38.7	9.9	74.0%	100.0%	74.0%
Moderate (AHI >= 15) with greater than 50% reduction in AHI	23.6	6.5	72.30%	95.8%	69.3%
Mild (AHI< 15) with greater than 50% reduction in AHI	10.3	3.5	62.99%	100.0%	62.9%

**Table 4:** Comparison of pre and post-treatment attribute scores The estimated means of the paired differences, 95% Confidence Intervals, and associated p-values are listed. *Indicates a p-value significant at the 0.05 level or greater.

Category	Mean difference (post – pre)	95% Confidence Interval	p-value
AHI (n=28)	-13.54	(-18.1, -9.0)	p<0.0001*
PSQI (n=24)	-1.42	(-3.0, 0.1)	p=0.07
ISI (n=25)	-4.00	(-6.2, -1.8)	p=0.001*
SSS (n=24)	-2.30	(-3.8, -0.8)	p=0.005*
FOSQ (n=27)	1.41	(0.6, 2.2)	p=0.001*

Similarly, QOL questionnaires showed significant improvement after treatment. The PSQI pretreatment mean score was 6.9 ± 4.6 and the posttreatment PSQI mean score was 4.8 ± 2.8. A PSQI score of 5 or more is an indicator of poor sleep. The use of MAD was associated with a PSQI reduction in 50% of subjects with a mean change of 1.4 ± 3.7. The pretreatment ISI mean was 12.0 ± 6.4 while the posttreatment mean was 7.6 ± 4.9, with a mean decrease of 4.0 ± 5.2. This ISI reduction occurred in 84% of subjects. The ISI index classifies patients with a score of 0 to 7 to have no clinically significant insomnia. The pretreatment FOSQ mean was 15.2 +/- 3.4; the posttreatment mean was 16.6 +/- 2.7; the mean change was 1.4. FOSQ was improved in 66.6% of subjects (note a higher FOSQ score indicates better functional status). The pretreatment SSS mean was 5.3 +/- 2.7, with a posttreatment SSS mean of 3.0 +/- 2.9, indicating a mean change of -2.3. The SSS reduction occurred in 69.6% of subjects. A lower SSS score indicates decreased snoring.

Paired t-tests were used to compare the difference between pretreatment and posttreatment scores for the following attributes: AHI, PSQI, ISI, SSS, and FOSQ. All differences were statistically significant with the exception of the change in PSQI. The AHI, ISI, and SSS decreased significantly after MAD use. FOSQ increased significantly with MAD use, indicating improved daytime function. Wilcoxon signed-rank tests were also calculated and yielded similar results. See Table 4 below. Change in AHI was greatest for those subjects with initial severe OSA (decrease by an average of 28.7 events/hour), moderate for those with initial moderate OSA (decrease by an average of 12.7 events/hour), and least for those with initial mild OSA (decrease by an average of 4.6 events per hour), as expected. Changes in daytime sleepiness were greatest for the moderate OSA group (statistically significant for SSS, but not ISI). Changes in quality of life (FOSQ) were also greatest for the moderate OSA group (p=0.02).

The MAD usage had a mean of 7.2 ± 0.9 hours, ranging from 5.0 to 8.4 hours for the study population. Patient-reported sleep time (n=28) averaged 6.7 ± 1.0 hours, ranging from 4.0 to 9.0 hours. MAD compliance overall according to DentiTrac (at least four hours a day, for five out of seven days a week) had a compliance rate of 93.6%, with a therapeutic efficacy mean of 54.6% to produce an MDA of 51.1%.

## Discussion

The present study demonstrates the therapeutic effectiveness of the Prosomnus [IA] MAD therapy calculated using MDA, a score that is determined from the product of the percent reduction in AHI and the patient adherence to treatment, measured by a compliance chip embedded in the MAD. The consideration of MDA as a basis for the comparison of therapeutic effectiveness allows the direct assessment of MAD therapy against the measured effectiveness of CPAP. For all patients in this study, the average MDA value was calculated to be 51.1% (Table 2). MDA values found in the medical literature for CPAP are similar at 50% [11,18]. While in some cases, MAD treatment may seem less efficacious than CPAP, the overall effectiveness of MAD is often superior to CPAP because of better adherence with treatment - findings consistent with our study and other published studies [18]. A common measure of success of oral appliance therapy is a 50% reduction in baseline AHI. In our study, 72.6% of subjects obtained this result, with an MDA in this group of patients of 71.1%.

OSA is associated with cardiovascular, metabolic, and neurocognitive disorders, which may improve with the treatment of the disease [19]. Despite CPAP’s efficacy in treating OSA, patient adherence to CPAP is low [18]. This adherence is dependent on initial perceptions of the treatment [20-21], which becomes mired in issues of discomfort with the apparatus, anxiety, inconvenience, and a lack of perceived benefit by users [22]. In contrast, the high percentage of MAD adherence found in this study corresponds with previous documented, if varied, patient preference for MAD therapy over CPAP [4,23-24].

Adherence to treatment is essential for the prevention of chronic diseases and improvement of quality of life. For patients using CPAP, the greatest benefits occur with at least six hours of use [25]. CPAP must be used throughout the night and especially during the second half of the night when there is generally a higher concentration of REM sleep and apneic events [18]. Thus, given the existing poor adherence with the four-hour per night minimum use criteria for CPAP compliance, MAD therapy becomes a more attractive treatment for patients due to longer periods of use in conjunction with higher nighttime adherence [26]. Our study supports the notion that compliance with MAD is superior to CPAP, as the period of mean MAD usage was 7.2 hours with an uncertainty of 0.9 hours and a range of 5.0 to 8.4 hours.

OSA is a lifetime illness, requiring patient adherence in the long term as well as the short term. Untreated OSA is associated with increased risks of myocardial infarction, stroke, reduced work performance, and increased occupational injuries creating an economic burden of 165 billion dollars a year in the United States [24,27]. Our study did not evaluate long-term adherence to treatment; however, published studies have found that long-term MAD adherence is comparable or better than CPAP adherence, with the four-year adherence of CPAP reported at 54% [22] and 10-year adherence of MAD therapy reported at 66% [28]. Furthermore, a two-year randomized study found no statistical difference between the proportion of patients obtaining successful treatment with CPAP compared to MAD therapy, with 50% of the patient population with severe OSA obtaining successful results with a MAD [24]. Severe sleep apnea patients are those most at risk for the adverse effects of OSA, and with failures of CPAP, this population can be lost to follow-up due to the inconvenience of CPAP. Thus, it is essential that issues of patient adherence are considered when evaluating which treatment options are appropriate, especially since MAD therapy has been demonstrated to be an effective treatment solution.

Long-term adherence was not evaluated in this study. Long-term oral appliance use can cause tooth movement, change in bite, and temporomandibular joint (TMJ) discomfort. However, several recent studies have shown that oral appliance therapy was associated with no significant tooth movement over a period of several years [29]. These side effects are rare and manageable with the proper selection of an oral appliance and patient follow-up [30].

The AHI is usually the main objective measure of the severity of sleep apnea and response to treatment. However, the AHI is not sufficient to describe either the physiological or behavioral impact of sleep apnea on health and daytime functioning. It should be noted that in the case of the three subjects in our study who had little or no improvement in their AHI scores on post-titration sleep studies, they did have an improvement in their QOL indicators. An evaluation of the raw data of the home sleep tests revealed fewer and shorter apneas in these subjects, despite a similar number of respiratory events. Given that the oxygen saturation nadir is associated with cardiovascular risk, reducing desaturations throughout the night even when more mild respiratory events persist (such as short hypopneas, rather than prolonged apneas) likely decreases cardiovascular risk. The AHI as a marker of severity of sleep apnea and as a measure of success of treatment is not ideal. Improvement in QOL scores noted in this study may explain why patients on treatment may report “feeling better,” even when the AHI is still in the mild or moderate range with the use of MADs.

## Conclusions

Given the very low risk of treatment complications with MADs, and the possibility of providing the treatment at low cost, otolaryngologists should offer MADs as a primary treatment choice to their patients with sleep apnea. Our study confirms that MADs can be used as a primary mode of treatment for patients with mild, moderate, and severe sleep apnea who refuse or cannot tolerate CPAP. Compliance was close to 100% in this study, with therapeutic outcomes similar to those described in published studies evaluating the effectiveness of CPAP.

## Appendices

Abbreviation: EFFECTS study = Efficacy and Effectiveness of the ProSomnus® [IA] Sleep Device for the Treatment of Obstructive Sleep Apnea study
